# An Investigation of Fiber Reinforced Chemically Bonded Phosphate Ceramic Composites at Room Temperature

**DOI:** 10.3390/ma11050858

**Published:** 2018-05-21

**Authors:** Zhu Ding, Yu-Yu Li, Can Lu, Jian Liu

**Affiliations:** 1 Guangdong Provincial Key Laboratory of Durability for Marine Civil Engineering, College of Civil Engineering, Shenzhen University, Shenzhen 518060, China; zding@szu.edu.cn (Z.D.); 13602500403@sina.com (Y.-Y.L.); 2 Infrastructure Construction Department, Shenzhen Polytechnic, Shenzhen 518055, China; lucan@szpt.edu.cn

**Keywords:** chemically bonded phosphate ceramic, fiber reinforced composites, PVA fiber, continuous carbon fiber sheet, acid activation, dynamic mechanical analysis

## Abstract

In this study, chemically bonded phosphate ceramic (CBPC) fiber reinforced composites were made at indoor temperatures. The mechanical properties and microstructure of the CBPC composites were studied. The CBPC matrix of aluminum phosphate binder, metakaolin, and magnesia with different Si/P ratios was prepared. The results show that when the Si/P ratio was 1.2, and magnesia content in the CBPC was 15%, CBPC reached its maximum flexural strength. The fiber reinforced CBPC composites were prepared by mixing short polyvinyl alcohol (PVA) fibers or unidirectional continuous carbon fiber sheets. Flexural strength and dynamic mechanical properties of the composites were determined, and the microstructures of specimens were analyzed by scanning electron micrography, X-ray diffraction, and micro X-ray computed tomography. The flexural performance of continuous carbon fiber reinforced CBPC composites was better than that of PVA fiber composites. The elastic modulus, loss modulus, and loss factor of the fiber composites were measured through dynamic mechanical analysis. The results showed that fiber reinforced CBPC composites are an inorganic polymer viscoelastic material with excellent damping properties. The reaction of magnesia and phosphate in the matrix of CBPC formed a different mineral, newberyite, which was beneficial to the development of the CBPC.

## 1. Introduction

Chemically Bonded Phosphate Ceramics (CBPCs) refers to a class of materials that are formed by a controlled acid-base reaction, which takes place at lower temperatures compared to sintered ceramics [1]. Although there are ionic, covalent, and van der Waals bonding in CBPC, the dominating are ionic and covalent bonding. CBPCs have high compressive strength, high resistance to high temperature and acidic environments. Their applications can fill the gap between the traditional hydraulic cements and the sintered ceramics [1,2]. CBPCs have many applications in the fields of structures, composites, and biomedical, high temperature environments. They are also utilized in radiation shielding, nuclear waste solidification and encapsulation [3]. Furthermore, the preparation of CBPC is castable, inexpensive, and environmentally friendly. One of the most studied CBPC materials is magnesium phosphate cement (MPC), which is formed by the reaction of magnesium and solution of alkali dihydrogen phosphate [4,5,6,7,8,9,10,11]. The microstructure of MPC based CBPC matrix consists of crystal (newberyite, struvite, and periclase) and amorphous phases [7,8,9]. These crystals behave like the fine aggregate in cement, which enhances the CBPC’s mechanical property and volume stability. Although CBPCs have good compressive properties but the tensile strength is relatively poor. Reinforcements, fibers or particles had been used to improve tensile properties, which greatly widened the applications of CBPC [12,13,14].

The other common type of CBPC is prepared from phosphoric acid and wollastonite [3]. Colorado et al. had carried out intensive research on this type of CBPC composite. Higher performance structure CBPC composites were manufactured with the reinforcements, for instance, nano-fibers, graphite nanoplatelets (GNPs) [15], glass fiber and carbon fiber [16]. The results from Colorado’s studies showed that the different fibers improved the properties of CBPC composites at different ranges. CBPC-GNPs showed an improved bending strength (more than 23 MPa without thermal treatment or aging) and high chemical stability below 600 °C. However, the interfaces between CBPC matrix and crystalline phases looks almost cracked, it should be conducted more research to improve the adhesion [15]. For the CBPC prepared by pultrusion method, the bending strength increased by a factor of 29 and 17 for pultruded carbon fiber and pultruded glass-fiber reinforcement CBPCs [16]. However, the pultrusion technique is complicated and higher cost.

In this study, we aimed to provide a simple method to prepare CBPC composite at room temperature, by using an aluminum phosphate binder rather than phosphoric acid. Aluminum phosphate is a type of inorganic binder usually used in refractory ceramics [17]. It is typical to harden aluminum phosphate by heating to elevated temperatures. After it is hardened, it becomes a solid material with high strength, high temperature stability, and good abrasion resistance [18]. However, aluminum phosphates are brittle materials which limit their applications in many important fields. To improve their ductility, researchers have attempted to develop phosphate-based composites using fibers. For example, a German company (Brunswick) has developed a phosphate based radome material [19]. Researchers in the USA have developed quartz fiber reinforced aluminum phosphate based composite materials, which have good mechanical and dielectric properties and can be solidified below 315 °C and still maintain good performance at 650 °C [20]. Russian researchers have explored various types of phosphates for creating phosphate-based composites. Among these phosphates, aluminum phosphate has shown excellent mechanical properties and high-temperature stability [21]. However, the preparation of these phosphate-based fiber composites has required elevated temperatures.

The current study investigates the flexural and dynamic mechanical properties of fiber reinforced CBPC composites produced at indoor temperatures. The CBPC matrix was composed of aluminum phosphate binder, metakaolin, and magnesia. Among them, the aluminum phosphate binder used in this study was synthesized from phosphoric acid and aluminum hydroxide, and magnesia was used as the curing agent. Two types of fiber, short polyvinyl alcohol (PVA) fiber, and continuous carbon fiber were used to make CBPC composites. Because the most building structures are under dynamic loads, both the flexural strength and the dynamic elastic modulus of the CBPC composites were measured. The microstructures of the composites were also examined. The reaction products and microstructure of both the CBPC matrix and the fiber composite were characterized by powder X-ray diffraction (XRD), electron scanning micrography (SEM), and micro X-ray computed tomography (XCT) analysis. The XCT analysis can present the distribution of fibers in CBPC composite, and leading to a better understanding of the combination status of fibers and matrix.

## 2. Materials and Methods

### 2.1. Raw Materials

The materials used in the experiment include: metakaolin (Jiaozuo, Henan Province, China), magnesia (Jimei Refractory Company, Zibo, Shandong Province, China), fine aggregate that was ISO standard sand (ISO 679, Amoy i standard sand Co., Ltd., Xiamen, Fujian Province, China), phosphoric acid (≥85%, Chengdu Kelong Chemical Reagent Factory, Chengdu, China) and aluminum hydroxide (Chengdu Kelong Chemical Reagent Factory, Chengdu, China). The chemical composition of the materials and the average particle size are given in Table 1. The technical parameters of PVA fiber (Kuraray Trading (Shanghai) Co., Ltd., Shanghai, China) are shown in Table 2. The dimensions and physical properties of continuous carbon fiber sheet (Nanjing Haituo Co., Ltd., Nanjing, China) are listed in Table 3.

### 2.2. Phosphate Solution Preparation

In this study, aluminum dihydrogen phosphate (Al(H_2_PO_4_)_3_) was used as the phosphate binder for making CBPC. It was prepared according to the following reaction equation:

Al(OH)_3_ + 3 H_3_PO_4_ = Al(H_2_PO_4_)_3_ + 3 H_2_O (1)

Al(OH)_3_ was evenly mixed with deionized water, then, the mixture was put into a magnetic stirrer and stirred in a water bath at 180 rpm. Next, H_3_PO_4_ was added into the mixture solution evenly and slowly when the water temperature reached 90 °C. Heating continued until water temperature reached 98 °C and was then held at that temperature. The mixture was stirred uniformly until the transparent substrate glue was observed. The stirring continued for one hour more for evaporating excess moisture. Finally, the phosphate binder solution was synthesized.

### 2.3. Test Specimen Preparation

The raw materials’ proportions used in the test specimens are listed in Table 4. The pure CBPC paste specimen was prepared by mixing phosphate solution, metakaolin, and magnesia, while the composites test samples with PVA fiber were made by mixing CBPC paste and PVA fiber. The blend mixtures were stirred evenly into an adhesive mixture. The mixtures were injected into a mold with the dimensions of 160 mm × 40 mm × 10 mm, then vibrated by using a cement mortar vibrator (ZP-5, Henan Zhongke Engineering & Technology Co., Ltd., Zhengzhou, China). After three days, the specimens were released and cured in laboratory conditions for 28 days (air temperature: 25 ± 2 °C; relative humidity: 60 ± 5%). The flexural strength was then tested by an MTS machine (WE-30B, Changchun Testing Machine Institute Co. Ltd., Changchun, China).

The procedure for the preparation of CBPC composites specimens with carbon fiber sheet is as follows: the CBPC paste was firstly mixed with the phosphate binder, metakaolin, and magnesia using the proportions as listed in Table 5. Then the composites were molded by using CBPC to paste the carbon fiber sheets layer-by-layer. The size of mold was 160 mm × 40 mm × 10 mm. A layer of freshly mixed CBPC paste was evenly placed on the bottom surface of the mold, and then a layer of carbon fiber sheet was laid on the freshly mixed CBPC paste. After that, a layer of freshly mixed CBPC paste was evenly placed on the carbon fiber sheet. The above procedure was repeated several times according to the number of layers of fiber sheets. After this assembly of the composites, the specimens were cured in laboratory conditions then the flexural strength was tested.

### 2.4. Flexural Strength Testing Method

**Figure 1:**
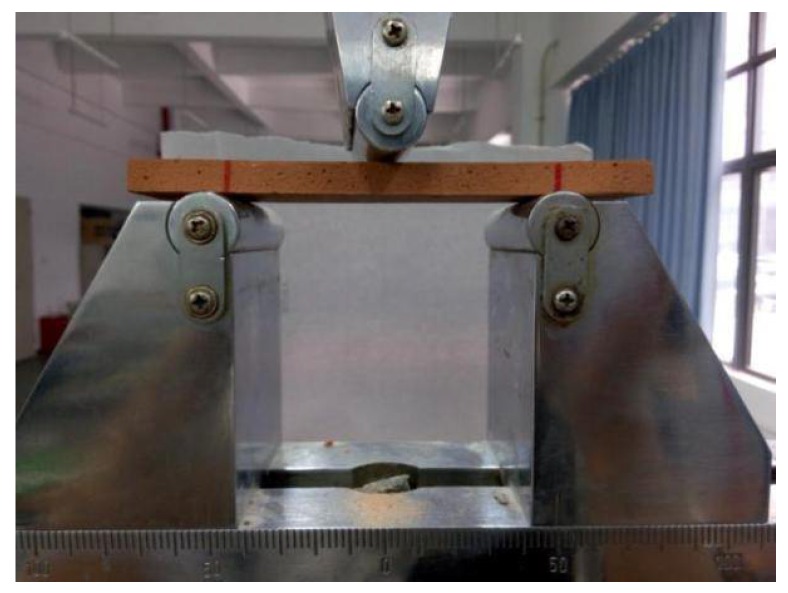
Specimen and three-point bending strength test setup.

The flexural strength of CBPC follows the fiber reinforced plastic flexural performance test method (GB/T 1449-2005). Figure 1 shows the test setup of the flexural test with a test specimen in place. The flexural modulus of fiber composites is calculated according to Equation (2).

(2) $$E_{f}=\frac{\Delta P\cdot L^{3}}{4b\cdot h^{3}\cdot \Delta S}$$

where *E_f_* is the flexural modulus of elasticity; *ΔP* is the load increment of the initial straight line on the load-deflection curve; *ΔS* is the deflection increment corresponding to the span midpoint specimen *ΔP*; *L* is the span during testing; *b* is the specimen width; *h* is the specimen thickness.

### 2.5. Dynamic Elasticity Testing Method

Dynamic mechanical analysis (DMA) has become a typical method to study the properties of polymer materials in recent years [22]. Phosphate binders are a type of inorganic polymer material so DMA is useful for its study. Physical parameters from DMA represent the dynamic elasticity and include the loss angle δ, storage modulus E′, and complex modulus E″. In this study, the dynamic viscoelasticity of fiber reinforced CBPC composite was studied by changing the size of the dynamic load and the type of fiber. Viscoelasticity refers to the characteristics of the microstructure of composites in response to external loads, reflecting the multiplicity of its motion units and the hysteresis of response to external loads.

Four groups of samples were prepared and cured according to the procedures mentioned in the Section-Test Specimen Preparation. The material mixtures used are listed in Table 6, and the specimen size was 10 mm × 10 mm × 40 mm. The hardened specimens surface were polished using 600 mesh sandpaper prior to DMA using the dynamic mechanical analyzer (DMA+1000, PerkinElmer, Shanghai, China). The loading range was 10 to 60 N, loading frequency was 2 Hz, and loading amplitude was 10 N.

### 2.6. Microstructure Analysis Method

To observe the microstructure of CBPC composite, powder X-ray diffraction (XRD) analysis was carried out using a D8 Advance (Bruker, Karlsruhe, Germany). The XRD used Cu Kα radiation (λ = 0.154 nm), had a tube voltage of 40 kV, and tube amperage of 10 mA. The scanning region was 10°~70° (2θ), and step width was 0.05° (2θ). A scanning electron microscope (SEM) (Quanta TM 250 FEG, FEI, Hillsboro, OR, USA) was used for the micromorphological observation of the CBPC specimens. The SEM observations included both pure CBPC paste and carbon fiber samples. The acceleration voltages were 5, 10 and 15 kV. Three-dimensional (3D) imaging micro-analysis was conducted for the PAV fiber samples by using Micro XCT-400 (Xradia Inc., Jena, Thuringia, Germany). The sizes of the samples were approximately 0.5 cm^3^. A large number of two-dimensional (2D) images were extracted, then Avizo software was used for reconstructing 3D images from these 2D images. The specimen with 1% PVA fiber was tested by this XCT analysis.

## 3. Results and Discussion

### 3.1. Flexural Strength of CBPC Composites with PVA Fiber

**Figure 2:**
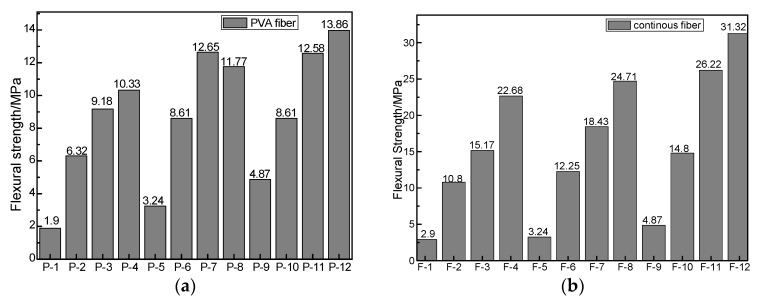
Flexural strength of CBPC composites with (**a)** PVA fiber and (**b)** continuous carbon fiber sheet.

The results of the flexural strength testing of CBPC composites with PVA fiber are shown in Figure 2a. It can be seen that when the content of the PVA fiber was the same, the flexural strength of the samples (samples P-1 to P-4) without magnesia was significantly lower than the comparable samples that contained magnesia. Thus, adding magnesia improved the flexural strength of phosphate-based fiber composites for the same curing ages. When the contents of PVA fiber were the same, the flexural strengths of the samples with 15% magnesia content (samples P-9 through P-12) were higher than that of other samples with the content of 12% magnesia (samples of P-5 through P-8). The CBPC sample mixture with a Si/P mole ratio of 1.2 and magnesia content of 15% developed excellent mechanical properties.

Compared to specimens having no PVA fiber, the flexural strength of CBPC composites with PVA fiber was seen to increase by about two or three times when the content of PVA fiber was 0.8%. With the increase of PVA fiber, the flexural strength of the specimen gradually increased. When the PVA fiber content was 1.5%, the flexural strength increased to 13.86 MPa.

In addition, PVA fiber can significantly improve the ductility of the CBPC material. When PVA fiber was not blended into the mixture, a typical brittle fracture occurred immediately when the displacement of the sample reached approximately 0.3 mm. When the PVA fiber content was 0.8% and 1.2%, the maximum displacements of the specimens were 4 mm and 6 mm, respectively. At a PVA fiber content of 1.5%, the displacement maximum was 6 mm. However, for this PVA fiber content of 1.5%, the failure mode changed from brittle failure to plastic failure. As this specimen had deformation before failure, a lot of fine cracks formed before final fracture. The test results indicate that phosphate-based short fiber composites have high ductility and provide good resistance to flexural deformation.

### 3.2. Flexural Strength of CBPC Composites with Continuous Carbon Fiber Sheets

The results of flexural strength testing of CBPC composites with carbon fibers are shown in Figure 2b. When the samples had the same number of layers of carbon fiber sheets but had different magnesia contents, the flexural strength values increased with the magnesia content. This increase of flexural strength with magnesia content is seen in the flexural strength of samples F-9 through F-12 being greater than their counterparts in samples F-5 through F-8, which in turn were greater than the comparable samples in F-1 through F-4. When the Si/P mole ratio was 1.2, and the magnesia content was 15%, the CBPC sample had the maximum flexural strength observed in this study.

When magnesia content was at the same level, adding the first layer of carbon fiber sheet gave a flexural strength that was three times that of the sample without the carbon fiber sheet. The single layer composite flexural strength was comparable to the strength of the composite sample with 1.5% PVA short fiber. The flexural strength increases with increasing the number of fiber sheet layers. When three layers of carbon fiber sheet were used, the flexural strength of the sample reached a maximum of 31.32 MPa. In general, phosphate-based continuous fiber composites have a better flexural resistance than the phosphate-based short fiber composites.

**Figure 3:**
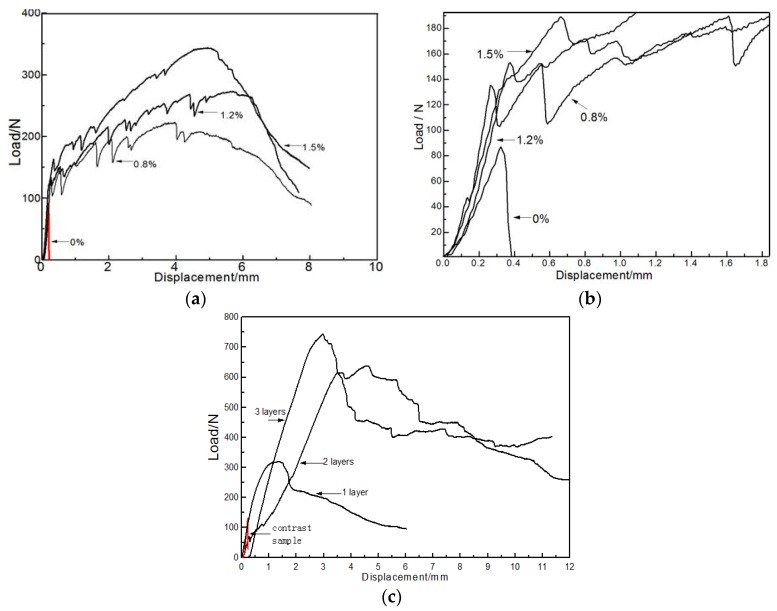
Loading-displacement curves for (**a**) CBPC composites with PVA fiber and (**b**) its partial enlargement; (**c**) CBPC composites with continuous carbon fiber sheets.

**Figure 4:**
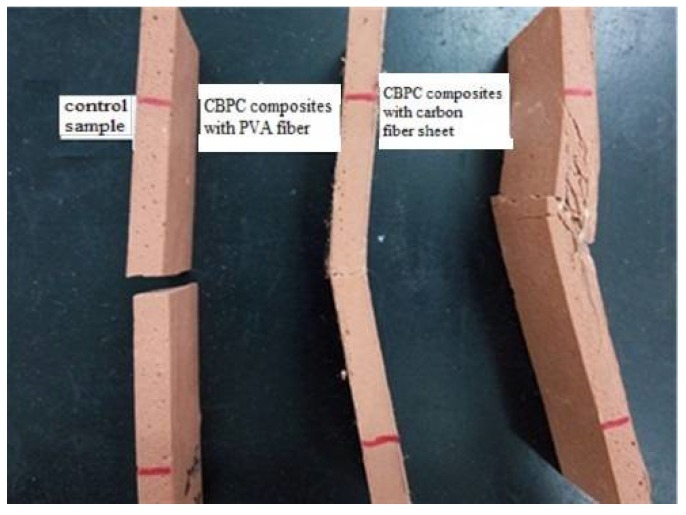
Failure modes of CBPC paste and CBPC composites with PVA fiber and continuous carbon fiber sheet.

Figure 3 displays the loading-displacement curves of both CBPC composites with short PVA fibers and carbon fiber sheets. Figure 4 shows the failure modes of the control sample and the CBPC composites with short PVA fibers and carbon fiber sheets.

**Figure 5:**
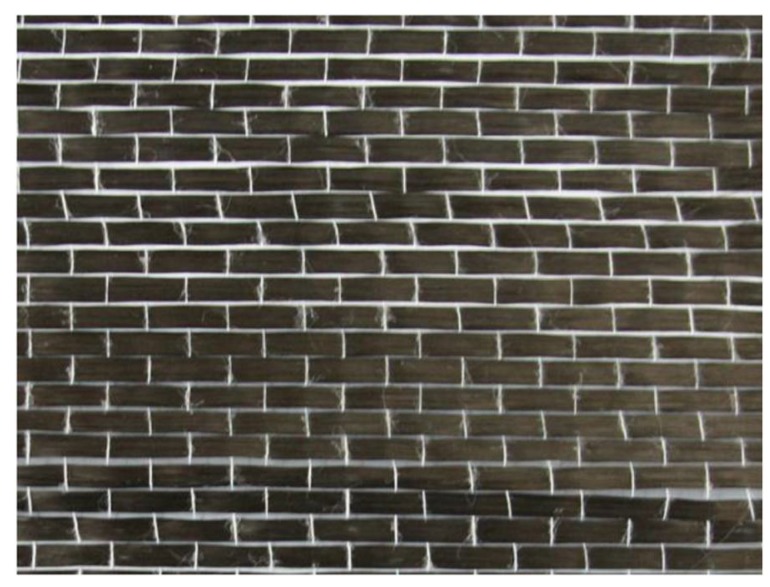
Continuous carbon fiber sheets showing spacing of fibers.

Fiber reinforced CBPC composites had an excellent flexural capacity in the tests. As well, fiber reinforced CBPC composites had better ductility compared to the pure CBPC matrix paste. Based on the experimental results, the modulus of elasticity of the sample without the fiber was 2.92 GPa. As the layers of the carbon fiber sheet were increased from 1 to 3, the elastic modulus significantly increased from 5.77 GPa for one layer, 8.31 GPa for two layers, and 10.88 GPa for three layers. As shown in Figure 4, the failure mode of the control specimen was the brittle fracture. Specimens with continuous fiber had a significant number of cracks in from the middle of the specimen that formed because of the constraint action of the continuous carbon fiber. There are small gaps in the carbon fiber sheets as shown in Figure 5 that allow more of the CBPC paste to penetrate. This additional penetration of CBPC likely provides strong bonding between the fiber and matrix. Similar to the short PVA fiber results, phosphate-based continuous fiber composites had high ductility and also gave good resistance to flexural deformation.

### 3.3. Dynamic Elastic Analysis of Fiber Reinforced CBPC Composites

The mechanical properties of polymer materials are affected by the structure, but also by the size of external forces, time, frequency, and temperature. During the dynamic elastic analysis, a sinusoidal load was applied to the phosphate composites. The material deformation occurred under the external load. The external force for the deformation is split into two parts. One part of the load is stored in the so-called storage modulus E’. Another part of the external force changes the molecular chain of the polymer which corresponds to the wasted loss modulus E”. The stress and strain of the material under the action of the sinusoidal load were measured by the sensors of the DMA instrument.

**Figure 6:**
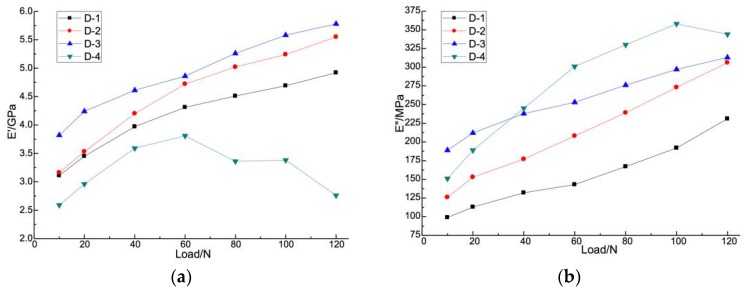

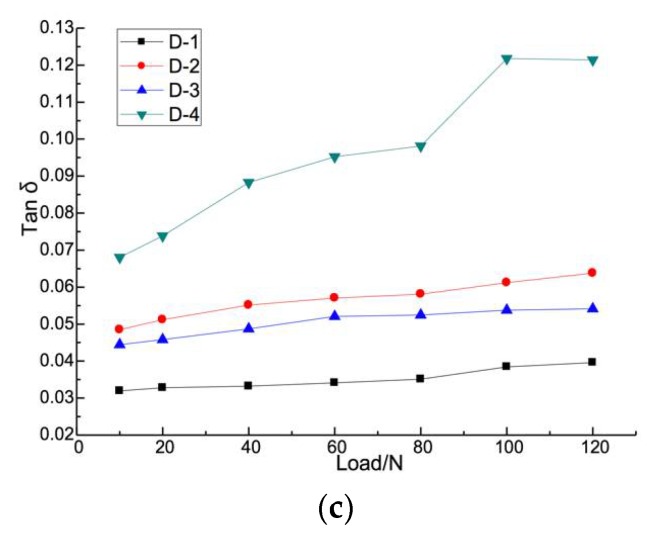
Dynamic elastic analysis of fiber reinforced CBPC composites showing the (**a**) storage modulus, (**b**) loss modulus, and (**c**) loss factor.

The results of dynamic elastic analysis of phosphate-based fiber composites are shown in Figure 6. It can be seen from Figure 6a that the storage modulus of sample D-4 increased at first and then decreased, while other samples showed an increasing trend with loading. Among all samples, the sample D-3 had the maximum storage modulus at all loads.

The magnitude of preloading in the dynamic load scanning is the same throughout. As the material is in the linear elastic range for the entire loading process, the dynamic storage modulus was only slightly changed. For polymer materials experiencing dynamic loading, the storage modulus of the material is the physical quantity related to the elastic deformation. Therefore, the CBPC specimen with short fibers could have increased internal friction of the molecular chain, slowing the slip phenomenon of the molecular chain, thus increasing both the stiffness and the storage modulus. This would explain the increased resistance to deformation. Also, the growth rate of the storage modulus of sample D-2 is faster than sample D-1 with loading. This difference indicates that the magnesia can effectively enhance the internal friction of the material, leading to the increase of damping, stiffness, and the storage modulus.

According to Figure 6b,c, the loss modulus and loss factor increased with dynamic loading. Loss modulus is also known as viscosity modulus, characterizing the ability to dissipate deformation energy and reflect the viscous nature of the material. The greater the loss modulus, the more energy the viscous deformation will lose, the closer the material is to the viscous material. The loss factor also represents the viscoelasticity of the material, proportional to elasticity and inversely proportional to elasticity. For sample D-4, the loss modulus and loss factor may increase so significantly as a result of the large amount of deformation seen in the sample and changes in molecular conformation. In the allowable load range for the test, the larger values of load are of more importance to flexural deformation resistance. So the sample D-4 is more reach to the viscous material. As these higher dynamic loads are reached, the loss modulus has increased, and it is seen specifically that the CBPC fiber composites have an increased resistance to viscous deformation. The loss moduli of the other samples only have low overall growth, and the growth rate is small as well relative to the CBPC fiber composites. So the CBPC fiber composites are closer to the viscous material compare to the CBPC paste.

The microstructure motion units respond to the magnitude of the external load. As well the response is sensitive to the static and dynamic components of the load. A static load is equivalent to applying a tension force to the element in advance of the dynamic load, thus reducing the response to the dynamic load. That is, a larger static load reduces the available dynamic load and therefore reduces the dynamic strain. As well, a larger static load results in a larger storage modulus. The dynamic storage modulus of the samples with continuous carbon fiber exhibit significant load sensitivity and have a strong non-linearity.

Compared with the pure CBPC matrix, CBPC fiber composites have excellent ability of resistance to deformation. Both CBPC with PVA fiber and continuous fiber composites have good resistance to elastic deformation, while the CBPC with continuous fiber composites has the strongest resistance to viscous deformation. With the alternating load between 10 N and 120 N, the specimens present viscoelastic mechanical properties that are related to elastic deformation. Increasing the magnesia content was efficient in improving the storage modulus and loss modulus of the viscoelastic materials.

### 3.4. XRD Analysis of the CBPC Paste

**Figure 7:**
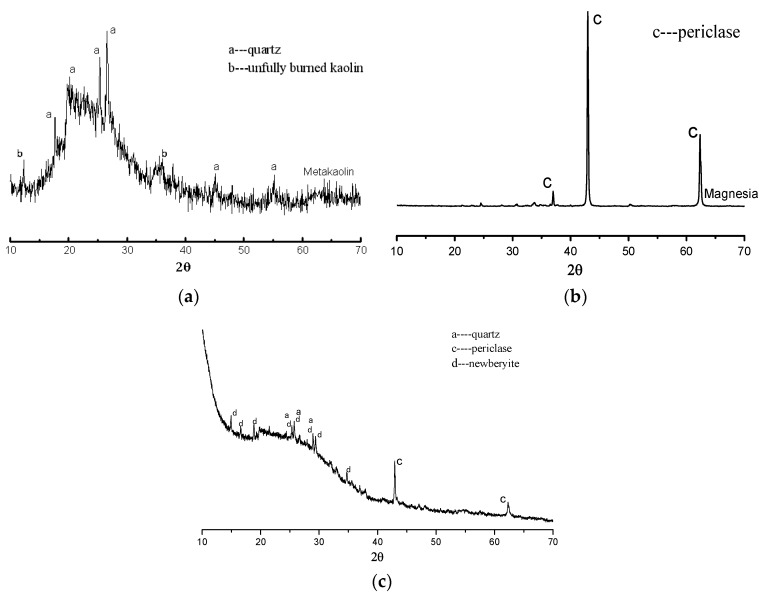
X-ray diffraction (XRD) spectra of (**a**) metakaolin, (**b**) magnesia, and (**c**) CBPC matrix.

The results of XRD analysis of metakaolin, magnesia, and CBPC matrix are shown in Figure 7. In Figure 7a, the metakaolin has an amorphous microstructure, and the amorphous peak formed between 15° and 35° (2θ). There were small characteristic peaks of quartz found in the spectrum. As well, the diffraction peaks of kaolinite were also seen. In Figure 7b, it is seen that there were sharp diffraction peaks in the XRD pattern of magnesia. The sharp peaks show that the magnesia had high crystallinity. Figure 7c shows the XRD pattern of the hardened pure CBPC matrix. This shows an amorphous peak of CBPC pastes formed between 15° and 35° (2θ), which means that the CBPC paste had an amorphous microstructure. In the XRD pattern, the diffraction peaks of quartz and periclase in CBPC are caused by the raw materials of metakaolin and magnesia that were used. However, a different mineral, newberyite (MgHPO_4_·3H_2_O), was also found in the hardened CBPC. This mineral was the reaction product of magnesia and phosphate solution. The newberyite mineral has fine needle-flake crystals [1,23,24] which may benefit the strength development of the CBPC matrix. According to the study of Wagh et al. [25], the reaction mechanism of CBPC may include a 3D polymeric network structure (–Si–O–Al–O–P–) formed by the reaction of phosphate and metakaolin. Magnesia cannot fully react with phosphate in the current mixtures, so the periclase and its XRD diffraction peaks remain in the hardened CBPC matrix. The diffraction peak of newberyite was in proportion to the content of magnesia in the sample. From the analysis, although magnesia was not involved in polymerization, it could react with the phosphate solution to form the newberyite crystal. The reaction equation of magnesia and phosphate solution can be written as follows:

(3) $$2\mathrm{MgO}+{\mathrm{Al}(H}_{2}{\mathrm{PO}}_{4}{)}_{3}+H_{2}O\to {\mathrm{MgHPO}}_{4}\cdot {3H}_{2}O+{\mathrm{AlPO}}_{4}\cdot {\mathrm{nH}}_{2}O$$

### 3.5. Microstructure of Fiber Reinforced CBPC Composites

**Figure 8:**
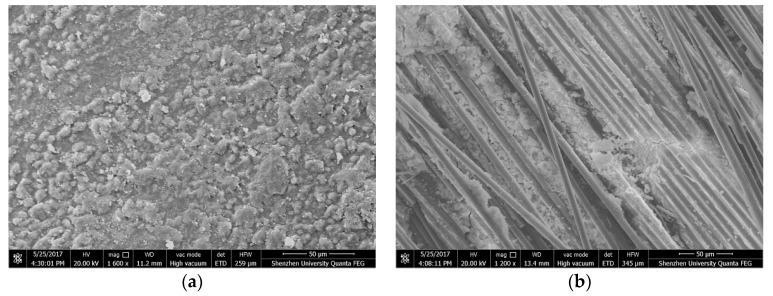
Electron scanning micrography (SEM) micrographs of fiber reinforced CBPC composites for (**a**) sample F-9 and (**b**) sample F-10.

To investigate the microstructure of CBPC composites, XCT, and SEM analysis were performed for the PVA and carbon fiber reinforced CBPC composites samples after curing for 7 days. Figure 8 shows the SEM pictures of carbon fiber CBPC composites of samples F-9 and F-10. Figure 8a illustrates that the hardened CBPC matrix has a dense microstructure. The reaction products are closely bonded together, and there are no visible original cracks in the matrix indicating that CBPC has a continuous microstructure. There are no visible crystals in the SEM picture, demonstrating that the produced crystal in the CBPC matrix is likely fine and uniformly distributed in the matrix.

**Figure 9:**
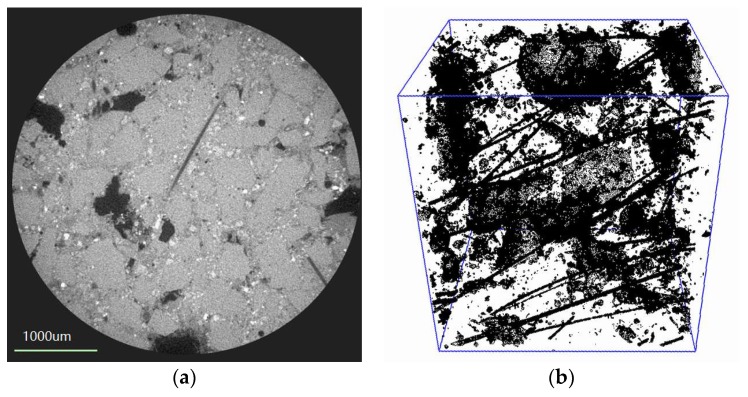
XCT analysis of fiber reinforced CBPC composites sample P-10 (**a**) XCT 2D plane figure; (**b**) XCT 3D reconstructed image.

Figure 9 shows the XCT analysis of PVA fiber CBPC composites sample F-10. Figure 9a displays one of the 2D plane figures scanned the sections by the XCT. The 2D figures were then used to complete the 3D reconstruction by Avizo software (Avizo 8.0, FEI, Hillsboro, OR, USA). The region size of the 3D reconstruction was 2.186 mm × 2.621 mm × 2.617 mm, and the 3D reconstructed image is shown in Figure 9b.

After the addition of PVA fibers and continuous carbon fibers, the flexural strength of CBPC was greatly improved. The increase of flexural strength can be partly revealed by the SEM and XCT analysis. In Figure 9b, the distribution of the continuous carbon fibers in the CBPC matrix is homogeneous. CBPC paste was bonded very well with the carbon fibers by permeating the spacing and getting through the continuous carbon fibers. Therefore, the matrix was able to transfer the load to the continuous carbon fibers without any interface problems, improving the mechanical property of the composites.

From Figure 9a, there are different gray values seen in the 2D XCT pictures. In this study, the gray value range is 0–255 in the 2D plane pictures from the XCT scanning. Different gray values represent different materials in the CBPC matrix. The gray values decrease from white to dark black corresponding to the magnesia particles, phosphate gel, PVA fiber, and air voids that were introduced during CBPC mixing. In Figure 9b, the 3D XCT reconstruction image shows the uniformly distributed PVA fibers with no mutual winding seen. The uniformed distribution occurs because phosphates are a good dispersing agent for the fibers. The good distribution of fibers provides an important “bridge effect” during loading which helps prevent crack propagation. As a result, the toughness of composites with continuous fibers was significantly enhanced.

## 4. Conclusions

In this study, fiber reinforced CBPC composites were fabricated at indoor temperatures. The mechanical property and microstructure of the CBPC composites were studied. From the experimental results, the following conclusions are made.

1. Both PVA fiber and continuous carbon fiber can significantly improve the toughness of CBPC. When 0.8 wt.% PVA fibers were used, the flexural strength was about twice that of the control sample, and the modulus of elasticity was 3.01 GPa. For 1.5 wt.% PVA fibers, the flexural strength increased to 13.86 MPa, and the modulus of elasticity was 4.2 GPa. For the composite with one layer of carbon fiber sheet, the flexural strength was 14.8 MPa, which is about three times that of the control sample. When two layers of fiber sheets were used, the modulus of elasticity was 5.77 GPa, which was far higher than the control sample and the composites sample with PVA fiber. For three layers of carbon fiber sheets, the flexural strength reached 31.32 MPa, and the modulus of elasticity was 10.88 GPa.
2. The results of dynamic mechanical tests indicate that CBPC fiber composites belong to the inorganic polymer viscoelastic materials as it had elastic deformation under alternating load. CBPC composites with short fiber had better resistance to elastic deformation than CBPC composites with continuous carbon fiber.
3. Magnesia can improve the mechanical property of the CBPC fiber composites. When the Si/P mole ratio of CBPC paste was 2 and magnesia content was 15%, the CBPC fiber composites had excellent flexural strength.
4. XRD analysis shows that CBPC matrix has an amorphous microstructure with residual quartz and magnesia in the matrix. A different mineral, newberyite, was observed to have been formed by the reaction of magnesia and phosphate. The SEM and XCT analyses show that the hardened CBPC matrix is dense, and the fiber can provide the “bridge effect” inside the fiber composite.

## Figures and Tables

**Table 1:** Chemical composition (wt.%) and average particle size of the main materials (μm).

	MgO	SiO_2_	Al_2_O_3_	CaO	Fe_2_O_3_	TiO_2_	P_2_O_5_	Average Particle Size (μm)
Metakaolin	0.06	55.06	44.12	0.17	0.76	0.81		8.13
Magnesia	83.18	6.42	1.19	6.467	1.278	1.25	1.25	46.65

**Table 2:** Technical parameters of polyvinyl alcohol (PVA) fiber.

Diameter (μm)	Elongation (%)	Length (mm)	Young’s Modulus (GPa)	Tensile Strength (MPa)	Density (g·cm^−3^)
40	12	7	41	1560	1.3

**Table 3:** Technical parameters of continuous carbon fiber sheet.

Thickness (mm)	Heavy (g/m^2^)	Tensile Strength (MPa)	Elastic Modulus (GPa)
0.11	200	3400	240

**Table 4:** Raw materials’ mixing proportions of chemically bonded phosphate ceramic (CBPC) composites with PVA fiber.

Sample Designation	Mix Proportion		
	Si/P Mole Ratio	Magnesia/Phosphate Solution (%)	PVA Fiber/Binder (%)
P-1	1.2	0	0
P-2	1.2	0	0.8
P-3	1.2	0	1.2
P-4	1.2	0	1.5
P-5	1.2	12	0
P-6	1.2	12	0.8
P-7	1.2	12	1.2
P-8	1.2	12	1.5
P-9	1.2	15	0
P-10	1.2	15	0.8
P-11	1.2	15	1.2
P-12	1.2	15	1.5

**Table 5:** Raw material mixing proportion of CBPC composites with continuous fiber.

Sample Designation	Mix Proportion		
	Si/P Mole Ratio	Magnesia/Phosphate Solution (%)	Number of Continuous Fiber Layers
F-1	1.2	0	0
F-2	1.2	0	1
F-3	1.2	0	2
F-4	1.2	0	3
F-5	1.2	12	0
F-6	1.2	12	1
F-7	1.2	12	2
F-8	1.2	12	3
F-9	1.2	15	0
F-10	1.2	15	1
F-11	1.2	15	2
F-12	1.2	15	3

**Table 6:** Material mixing proportion of the samples for dynamic mechanical analysis (DMA) by dynamic mechanical analyzer.

Sample Designation	Mix Proportion		
	Si/P Mole Ratio	Magnesia	Fiber Content
D-1	1.2	0	-
D-2	1.2	15%	-
D-3	1.2	15%	1.5% PVA fiber
D-4	1.2	15%	one layer of carbon fiber sheet
